# Efficacy of tranexamic acid versus placebo in reducing blood loss during burn excisional surgery: a multi-center, double-blind, parallel, randomized placebo-controlled clinical trial (TRANEX)

**DOI:** 10.1186/s13063-024-08332-1

**Published:** 2024-08-02

**Authors:** R. K. Gigengack, J. Slob, A. Meij-de Vries, E. Bosma, S. A. Loer, J. S. H. A. Koopman, C. H. van der Vlies

**Affiliations:** 1 Department of Intensive Care, Amsterdam UMC Location Vrije Universiteit, Amsterdam, De Boelelaan 1117, Amsterdam, the Netherlands; 2 Department of Anesthesiology, Amsterdam UMC Location Vrije Universiteit, Amsterdam, De Boelelaan 1117, Amsterdam, the Netherlands; 3 Departments of Trauma and Burn Surgery, Maasstad Hospital, Rotterdam, the Netherlands; 4 Department of Surgery, Red Cross Hospital, Beverwijk, The Netherlands; 5 Department of Surgery, Martini Hospital, Groningen, The Netherlands; 6 Department of Anesthesiology, Maasstad Hospital, Rotterdam, the Netherlands; 7 Trauma Research Unit Department of Surgery, Erasmus MC, Rotterdam, the Netherlands

**Keywords:** Tranexamic acid, Burns, Excisional surgery, Blood loss, Fibrinolysis

## Abstract

**Background:**

Despite an increase in knowledge, blood loss during burn excisional surgery remains a major challenge and is an independent predictor of mortality. During burn surgery, limited measures are available to control the bleeding. Increased fibrinolysis could be one of the contributing factors of blood loss during burn excisional surgery. Tranexamic acid inhibits the fibrinolytic response, and a small body of evidence shows positive effects of tranexamic acid on the volume of blood loss.

**Methods:**

The main objectives of this study are twofold, (1) to investigate whether tranexamic acid reduces blood loss and (2) to investigate the changes in coagulation after burn trauma and during burn excisional surgery. This study is a multicenter double-blind randomized clinical trial in patients scheduled for burn excisional surgery within the Dutch burn centers. All adult patients scheduled for burn surgery with an expected blood loss of ≥ 250 are eligible for inclusion in this study. The study is powered on a blood loss reduction of 25% in the intervention group. In total, 95 subjects will be included. The intervention group will receive 1500 mg tranexamic acid versus placebo in the other group. Primary endpoint is reduction of blood loss. Secondary endpoints include occurrence of fibrinolysis during surgery, graft take of the split skin graft, and differences in coagulation and blood clot formation.

**Discussion:**

This protocol of a randomized controlled trial aims to investigate the efficacy of tranexamic acid in reducing blood loss during burn excisional surgery. Furthermore, this study aims to clarify the coagulation status after burn trauma and during the surgical process.

**Trial registration:**

EudraCT: 2020-005405-10; ClinicalTrial.gov: NCT05507983 (retrospectively registered in August 2022, inclusion started in December 2021).

## Administrative information

Note: the numbers in curly brackets in this protocol refer to SPIRIT checklist item numbers. The order of the items has been modified to group similar items (see http://www.equator-network.org/reporting-guidelines/spirit-2013-statement-defining-standard-protocol-items-for-clinical-trials/).

**Table:** 

Title {1}	Efficacy of tranexamic acid versus placebo in reducing blood loss during burn excisional surgery, a multi-center, double blind, parallel, randomized placebo-controlled clinical trial. (TRANEX)
Trial registration {2a and 2b}.	EudraCT: 2020-005405-10 ClinicalTrial.gov: NCT05507983 (retrospectively registered in august 2022, inclusion started in December 2021)
Protocol version {3}	First approved version by the ethical committee: protocol version 1.2, February 2021, current version: 2.2.2 September 2023. Two amendments, June 2023, and November 2023. Amendment June 2023: change of the study end date due to slow recruitment; Amendment November 2023: change of structure of the blood samples and revisions considering the (S)AE reporting. In version 1.2, additional blood samples to investigate the burn coagulopathy were taking in all patients. In version 2.2.2 only patients included in the Maasstad Hospital will undergo additional blood analysis.
Funding {4}	The study is funded by the Dutch Burn Foundation, WO 19.102, (Nederlandse Brandwonden Stichting, Zeestraat 29, 1941 AJ Beverwijk, the Netherlands) Werfen B.V. will provide half of all ROTEM-consumables used in this study (Werfen B.V., Burfsteen 6, 4815PL Breda, the Netherlands).
Author details {5a}	R.K. Gigengack^1,2,3^, J. Slob^3^, A. Meij-de Vries^4^, E. Bosma ^5^, S.A. Loer^2^, J.S.H.A. Koopman^6^, C.H. van der Vlies^3,7^ ^1^ Amsterdam UMC location Vrije Universiteit, Amsterdam, Department of Intensive Care, De Boelelaan 1117, Amsterdam, the Netherlands ^2^ Amsterdam UMC location Vrije Universiteit, Amsterdam, Department of Anesthesiology, De Boelelaan 1117, Amsterdam, the Netherlands ^3^ Departments of Trauma and Burn surgery, Maasstad Hospital, Rotterdam, the Netherlands ^4^ Department of Surgery, Red Cross Hospital, Beverwijk, The Netherlands ^5^ Department of Surgery, Martini Hospital, Groningen, The Netherlands ^6^ Department of Anesthesiology, Maasstad Hospital, Rotterdam, the Netherlands ^7^ Trauma Research unit Department of surgery, Erasmus MC, Rotterdam, the Netherlands
Name and contact information for the trial sponsor {5b}	Prof. dr. E. Middelkoop Association of Dutch Burn Centres (ADBC) Postbus 1015 1940 EA Beverwijk
Role of sponsor {5c}	The sponsor of this study has no role in the study design, analysis or decision of the submission for reporting.

## Introduction

### Background and rationale {6a}

Major burn trauma involves a complex interplay between activation of coagulation and activation of the immune system [1]. Although the underlying pathophysiology behind these processes is not completely understood, more knowledge became available in recent years. Following severe burn injury, the patient’s coagulation status may change considerably as a response to the systemic inflammatory response and as a result of interventions such as hemodilution and during fluid resuscitation and excisional surgery [2]. The development of coagulopathy has been identified as a risk factor for increased morbidity and mortality after major burn trauma [3, 4]. Despite an increase in knowledge, blood loss during burn excisional surgery remains a major challenge and is an independent predictor of mortality [5]. During burn excisional surgery, limited interventions are available to control the bleeding during surgery. For example, the use of topical adrenalin and topical tranexamic acid and the use of a tourniquet have been described in literature [6]. Despite these techniques, blood loss is still substantial [7].

Cell salvage is a well-known method to reduce allogenic blood transfusion during high blood loss surgery. The use of cell salvage is feasible in burn patients and up to 25% of blood loss can be recovered. However, despite strict sterile handling of shed blood, the erythrocyte concentrate showed substantial bacterial contamination [8].

Reducing blood loss and tailoring blood product administration may improve patient outcome and reduce morbidity and mortality associated with transfusion and blood loss [3]. Therefore, well-functioning coagulation is of the upmost importance.

Normally, coagulation is a balanced process in which there is simultaneous coagulation and fibrinolysis. However, several studies describe a disruption of the immune system and coagulation comparable to changes after major trauma [3, 9, 10]. Marked increase of activation in coagulation paradoxically coexisted with an increase of fibrinolysis [4, 9, 11].

The increased fibrinolysis could be one of the contributing factors of blood loss during burn excisional surgery as fibrinolysis is seen in burn patients [9]. Furthermore, moderate to severe disturbance of the fibrinolytic system is seen in > 50% of all trauma patients [12].

Tranexamic acid inhibits the fibrinolytic response by inhibiting the conversion of plasminogen to plasmin. Normally, plasmin breaks down fibrin to fibrin degradation products thereby weakening the clot. By lowering the concentration of plasmin by the administration of tranexamic acid, the breakdown of the cloth will be inhibited. There is some body of evidence suggesting the beneficial effect of tranexamic acid on the perioperative blood loss and transfusion [13–15]. A small blinded randomized prospective study found a reduction of 36% in blood loss in 27 tangential burn excisions by administrating 20 mg/kg tranexamic acid intravenous [13], while a small RCT with 50 patients performed in India showed a reduction of 41% of perioperative blood loss with 15 mg/kg tranexamic acid intravenous [14]. Furthermore, a retrospective study reported a 30% decrease in transfusions during burn excisional surgery by administration of 10 mg/kg tranexamic acid intravenous followed by a continuous infusion of 1 mg/kg/h [15]. However, no study investigated the fibrinolytic state of burn patients during burn excisional surgery. There is sufficient evidence available for the safety of the use of tranexamic acid in other patient categories. Several large studies, CRASH-II and III trial [16, 17], WOMAN-trial [18], and a recent retrospective case–control study [19], have investigated possible thromboembolic complications of the use of tranexamic acid in trauma patients, during orthopedic surgery and women with postpartum hemorrhage, and reported no higher incidence in any complication in the tranexamic acid group.

The perioperative monitoring of coagulation and especially fibrinolysis using classic hemostatic tests is difficult [3]. More real-time test like thrombelastography (TEG) or rotational thromboelastometry (ROTEM) could give insight especially in the state of the fibrinolytic system [1, 10]. Schaden et al. reported a reduction in transfusion during burn excisional surgery by using a coagulation management protocol including ROTEM [9, 20]. While ROTEM is promising, ROTEM could miss moderate fibrinolysis [12]. To further study the fibrinolytic system, more in-depth laboratory tests are needed. For example, plasmin-α2-antiplasmin complex is a separate test and is a more sensitive parameter to investigate the occurrence of fibrinolysis.

The main goal of this study is twofold. First is to investigate the ability of tranexamic acid to reduce blood loss and blood transfusion during burn excisional surgery. Second is to investigate the occurrence and magnitude of fibrinolysis during burn excisional surgery.

### Objectives {7}

#### Research hypothesis

- Tranexamic acid reduces the volume of blood loss during burn excisional surgery, irrespectively of the presence of hyper fibrinolysis
- During burn surgery, a significant effect of hyper fibrinolysis is present due to the stress of burn trauma

#### Primary objective

- To determine whether tranexamic acid reduces the volume of blood loss compared to placebo in patients undergoing burn excisional surgery
- To determine the occurrence and extent of fibrinolysis during burn excisional surgery

#### Secondary objective

- To investigate the influence of tranexamic acid of the graft take/loss

### Trial design {8}

The TRANEX-study is designed as a double-blind, placebo-controlled, randomized superiority trial with two parallel groups and primary endpoint of blood loss during surgery. In total, 95 patients will be allocated to either tranexamic acid or placebo. A variable block randomization will be used stratified by center in which the patient is enrolled. The investigators, participants, and surgeon as well as the statistical procedure will be blinded. Analysis will be performed with the intention to treat principle.

## Methods: participants, interventions, and outcomes

### Study setting {9}

This multicenter study is a collaboration between all dedicated burn centers in the Netherlands: Maasstad Hospital in Rotterdam, Martini Hospital in Groningen, and the Red Cross Hospital in Beverwijk. Participant recruitment will be from patients scheduled for burn excisional surgery with an expected blood loss of > 250 ml. The surgery and study procedures will be performed in the designated center in which the patient is scheduled for surgery.

### Eligibility criteria {10}

#### Inclusion criteria

- Patients scheduled for burn excisional surgery
- An expected blood loss of ≥ 250 ml based on the estimation by the performing surgeon based on:
  - ◦ ≥ 2% body surface area excision planned (100–200 cc/%BSA excised, based on retrospective data from Burn Centre Rotterdam)
  - ◦ Operation technique used (some techniques show more blood loss than others)
  - ◦ Time after burn trauma (i.e., expected healing and subsequently the extent of bleeding) 
-  ≥ 18 years old
- Informed consent of patient or legal representative
- Patients or legal representative should have enough knowledge of Dutch to provide informed consent

### Exclusion criteria

- Patients with a recorded coagulopathy in their history
- The use of anticoagulants (except acetylsalicylic acid or (intensive) prophylaxis low-molecular weight heparin > 12 h before surgery)
- Severe kidney failure (creatinine > 500 μmol/L)
- Allergy for tranexamic acid
- Diagnosis of acute venous/arterial thrombosis within 3 months before inclusion
- Diffuse intravascular coagulation (based on the DIS-score > 5)
- Pregnancy
- Active breastfeeding
- History of epilepsy

### Who will take informed consent? {26a}

The main physician will ask the patient’s consent for a member of our study team to approach the patients with information regarding this study. The study team consist of research nurses and junior and senior investigators; the whole team is trained by the Good Clinical Practice guidelines (ICH E6 GCP): GCP-WMO or (e)BROK©.

The member will orally explain the study, provide additional written information, and answer possible questions; hereafter, there is a reflection time of a minimum of 24 h. After at least 24 h, the study member returns to the possible participant, and there is a possibility to discuss the study and possible questions. If the participants agree to participate in this study, written consent will be obtained. Informed consent will be gained from the participants, provided that she/he is able to understand the patient information and consider the pros and cons of study participation. If the participants can provide informed consent for the operation and anesthesia, the patient is able to consider participation and give informed consent for this study. In participants who are cognitively impaired or sedated in the ICU department, a legal representative will be asked to consider study participation and give informed consent. The participants will be asked for written informed consent when they regain the ability to provide informed consent.

### Additional consent provisions for collection and use of participant data and biological specimens {26b}

This is not applicable for the samples taken for the main purpose is to answer the research question. Participants are specifically asked for permission to store the samples for 5 years for future research.

## Interventions

### Explanation for the choice of comparators {6b}

At the moment, there is limited body of evidence suggesting the beneficial effect of tranexamic acid on the perioperative blood loss and transfusion [13–15]. However, there is insufficient evidence as there are no large randomized controlled trials available to make a definite conclusion. Therefore, tranexamic acid will be compared to placebo to evaluate the efficacy of tranexamic acid.

### Intervention description {11a}

**Fig. 1:**
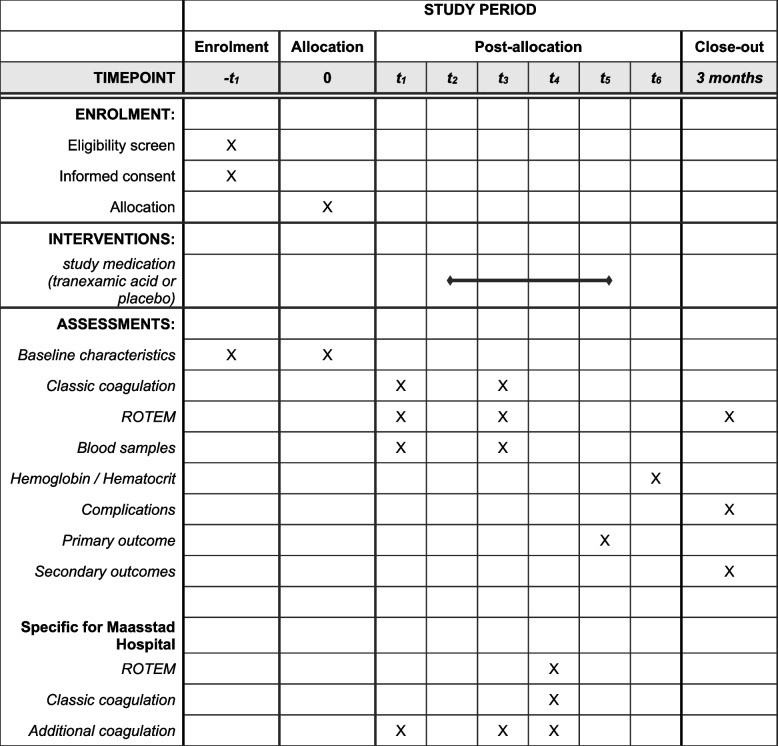
SPIRIT figure, schedule of enrolment, interventions, and assessments. -t_1_: > 24 h before surgery; t_1_: pre-surgery; t_2_: after anesthesia induction but before incision; t_3_: 1 h after incision; t_4_: during closure if total surgery time > 1.5 h; t_5_: end of surgery; t_6_: > 6 h after finishing surgery. *Blood samples*: hemoglobin, electrolytes (sodium/potassium), kidney function (urea/creatinine), albumin. *Basic coagulation*: INR, APTT, thrombocytes and fibrinogen. *Additional coagulation*: plasminogen activator inhibitor, D-dimer, global fibrinolysis, thrombin generation fVII, von-Willebrand Factor and anti-thrombin III and stored serum. *Complications*: cardiopulmonary or neurologic complication within the 3 months after the study period. ROTEM, rotational thromboelastometry

Patients will be prepared for surgery and anesthetized following the current standard of care. Both the placebo as the tranexamic acid will consist of an infusion bag of a total volume of 100 ml and will be indistinguishable for study personnel. See Fig. 1 for the complete timeline of study procedures. During induction of anesthesia, but before incision, the study medication will be administered over a period of 15 min, not surpassing the maximum delivery rate of tranexamic acid of 100 mg/minute. The intervention group will receive 1500 mg tranexamic acid intravenous. The placebo group will receive a sodium chloride 0.9% bolus.

Blood loss is difficult to measure; therefore, we will have a dedicated person available to monitor and register blood loss and measure the size of the excision. To determine the size of the excision, the excision site will be measured using a ruler. To register blood loss, we will use two methods. Firstly, intraoperative blood loss will be estimated by measuring the volume of blood collected in suction canisters, the weight of blood-soaked material (gauzes, pads, etc.), and the volume of adrenalized saline and subtracting the dry weight of these materials and the volume of adrenalized saline used for cleaning the surgical site. Secondly, we will calculate blood loss based on the preoperative hematocrit, postoperative hematocrit, and the intraoperative transfusions using the formula proposed by Camarase et al. in 2006 [21]. We will use this method because intraoperative transfusions are incorporated in the formula [21].

$$\begin{array}{c}\mathrm{Total}\;\mathrm{blood}\;\mathrm{loss}\;(\mathrm{ml})\;=\;\mathrm{total}\;\mathrm{red}\;\mathrm{cell}\;\mathrm{loss}\;(\mathrm{ml})\;/\;(\mathrm{hematocrit}\;\mathrm{before}\;\mathrm{surgery}\;-\;\mathrm{hematocrit}\;\mathrm{after}\;\mathrm{surgery})\\\mathrm{Total}\;\mathrm{red}\;\mathrm{cell}\;\mathrm{loss}\;(\mathrm{ml})\;=\;\mathrm{accepted}\;\mathrm{red}\;\mathrm{cell}\;\mathrm{loss}\;(\mathrm{ml})\;+\;\mathrm{volume}\;\mathrm{of}\;\mathrm{transfused}\;\mathrm{red}\;\mathrm{blood}\;\mathrm{cells}\\\mathrm{Accepted}\;\mathrm{red}\;\mathrm{cell}\;\mathrm{loss}\;=\;\mathrm{Estimated}\;\mathrm{volume}\;\ast\;(\mathrm{hematocrit}\;\mathrm{before}\;\mathrm{surgery}\;-\;\mathrm{hematocrit}\;\mathrm{after}\;\mathrm{surgery})\\\mathrm{Estimated}\;\mathrm{volume}\;\mathrm{in}\;\mathrm{men}\;=\;\mathrm{weight}\;(\mathrm{kg})\;\ast\;70\;\mathrm{in}\;\mathrm{women}\;\mathrm{weight}\;(\mathrm{kg})\;\ast\;65\;(\mathrm{formula}\;\mathrm{of}\;\mathrm{Moore})\end{array}$$

Blood sample will be taken at three time points during the intervention: before surgery, 1 h after incision, and 6 h after finishing surgery. The blood samples consist of the following analysis: before surgery: hemoglobin, electrolytes (sodium/potassium), kidney function (urea/creatinine), albumin, ROTEM, basic coagulation test (INR/APTT/thrombocytes/fibrinogen); during surgery: ROTEM; after surgery: hemoglobin and hematocrit. In addition to the above-described analysis, additional blood samples will be taken in all patients studied in the Maasstad Hospital. When surgery is ≥ 1.5 h, an additional ROTEM will be determined at the end of surgery. Furthermore, in the Maasstad Hospital, additional samples will be collected to investigate the burn coagulopathy during the perioperative period (i.e., global fibrinolysis, D-dimer, anti-thrombin III, etc.).

### Criteria for discontinuing or modifying allocated interventions {11b}

In patients with impaired renal function (creatinine > 120 μmol/L), the dose will be reduced to 1000 mg tranexamic acid.

### Strategies to improve adherence to interventions {11c}

As this is a single intervention study, the medication will be administered once during surgery, and no measures will be taken to improve adherence.

### Relevant concomitant care permitted or prohibited during the trial {11d}

Correction of coagulation and transfusion will be at the discretion of the attending anesthesiologist. The laboratory results taken for this study will not be available for the attending anesthesiologist. Furthermore, the perioperative surgical hemostatic measures (i.e., adrenaline-soaked gauzes and subcutaneous adrenaline injection) will be at de discretion of the performing surgeon appropriate to the local protocol. As such, the concomitant care remains similar between groups.

### Rescue medication

In the event of massive, uncontrollable bleeding, leading to multiple transfusion and the need for correction of the coagulation, patients will be given 1000 mg of tranexamic acid, regardless of the original randomization.

### Provisions for post-trial care {30}

After completing the trial, participants will be treated with the standard of care.

### Outcomes {12}

Primary outcome:

- The volume of blood loss in milliliter/% BSA excised measured during surgery

Secondary outcome:

- The occurrence and magnitude of fibrinolysis/coagulopathy during burn excisional measured by, among others, ROTEM-analysis, the measurement of global fibrinolysis and D-dimer
- Graft take of the split skin graft on the area of excision measured in the percentage of vital graft 5 days surgery
- Hospital mortality
- Length of (ICU) stay
- Cardiopulmonary (i.e., arterial embolism)/ neurologic complication (i.e., stroke) (at 3 months after the study procedure)
- Need for escape medication (tranexamic acid 1000 mg) in case of uncontrolled bleeding
- The need for red blood cell transfusion peri-procedural

### Participant timeline {13}

When a potential participant is scheduled for burn excisional surgery, the patient is informed about the study and informed consent is taken. See Fig. 1 for the complete timeline for the participant. Informed consent is usually taken in the 2-3 days before surgery. After consent, the patients are randomized. A blood sample is taken before surgery to establish a baseline. During surgery, two more blood sample will be taken, and one blood sample will be taken 6–12 h after surgery. Hereafter, the study procedure is finished for the patient. After 3 months, the patient will be contacted to inform whether cardiopulmonary of neurologic complications have occurred.

### Sample size {14}

In a small retrospective group of 109 patients (unpublished) with ≥ 250 ml blood loss from Burn Centre Rotterdam, we found a mean blood loss of 1.01 ± 0.56 ml/cm^2^ or 194 ± 81 ml/% BSA excised. In a small blinded RCT study, Jennes et al. found a reduction of 36% in blood loss in 27 tangential burn wound excisions when administering tranexamic acid [12]. A small RCT with 50 patients performed in India showed a reduction of 41% blood loss in burn patients when using tranexamic acid [13]. Orthopedic literature shows a somewhat more limited blood loss reduction of approximately 25% with the use of 1–2 g of tranexamic acid during surgery [20, 22, 23]. Therefore, based on a mean blood loss of 194 ml/% BSA excised with an SD of 81 ml, demonstrating a 25% reduction in blood loss with type I and type II error rate of 5% and 20% respectively, we require 45 patients per arm, for a total of 90 patients. Taking a dropout rate of 5% into account, we aim to include 95 patients in our study.

### Recruitment {15}

All patients scheduled for surgery in one of the study sites will be screened whether eligible for inclusion. The screening will be performed by the patient’s main physician, and after eligibility is confirmed, a member of the study team will approach the patient. The enrollment period will extent over 24 months or longer if needed. The enrollment rate will be dependent on the study site. We expect a combined (multi-center) recruitment rate of 5 patients per month. To monitor and improve recruitment, patients eligible, but not recruited for the study, will be identified and analyzed to determine the reason for not participating in this study.

## Assignment of interventions: allocation

### Sequence generation {16a}

Participants will be randomized into two groups (1:1 allocation ratio) using the randomization tool in Castor EDC (Castor EDC, Ciwit BV, Amsterdam, The Netherlands). Variable block randomization will be used, stratified by study site. The block size will be set to 2, 4, and 6 as the possible minimum number of inclusions per center is 16. The study team is always concealed of the sequence of randomization, current randomization or previous randomizations.

### Concealment mechanism {16b}

This is not applicable, as the randomization is done digitally in the Castor EDC (Castor EDC, Ciwit BV, Amsterdam, The Netherlands) environment without the ability to see the randomization process by the study team.

### Implementation {16c}

The allocation sequence is generated on demand during the randomization process in Castor EDC (Castor EDC, Ciwit BV, Amsterdam, The Netherlands) and are automatically assigned to the randomized participants.

## Assignment of interventions: blinding

### Who will be blinded {17a}

The randomization result will be held in the Castor EDC program which remains hidden during the whole study procedure and data analysis. As such, the patients, the study team, the treating physician, the operation team (i.e., surgeon/anesthesiologist), and the outcome assessors will be blinded. The unblinding will be performed after completion and data analysis. Via a separate login in Castor EDC, the pharmacy can view the result of the randomization and prepare the study medication. The study medication is a 100-ml clear NaCl 0.9% solution with no visual difference between the placebo of the tranexamic acid group. As such, the blinding remains intact for the team.

### Procedure for unblinding if needed {17b}

Emergency unblinding will only be performed in case of any adverse or serious adverse event which is unexpected and out of proportion with the standard of care. Unblinding will be performed by a member of the primary research team. All involved research member can reach the member of the primary research team 24/7.

## Data collection and management

### Plans for assessment and collection of outcomes {18a}

By standardizing the study procedure using a Standard Operation Procedure (SOP) the data collection, measurement and timing of blood samples will be equal in all study sites. The study team will be trained to perform and guide the perioperative study procedure. The measurement of blood loss is standardized and twofold to create higher accuracy. All data will be collected digitally.

### Plans to promote participant retention and complete follow-up {18b}

This is not applicable. The whole study period will be performed while the participants are in the hospital, and participants can therefore be directly monitored.

### Data management {19}

All data will be entered and stored digitally in the Castor EDC environment. Castor EDC provides account/password-protected access to the database. The study team and principal investigator can assign different rights to each individual, for example to read and write. When creating a new record, Castor EDC will automatically create an ID for the patient based on inclusion site, number of inclusions of that specific site, and treatment arm. This ID will be used for all future reference with data and blood samples regarding this patient. The handling of personal data will comply with the Dutch Personal Data Protection Act and Data will be stored for 15 years.

### Confidentiality {27}

The Castor EDC will be used to collect and store all data. Castor EDC is compliant with the ICH E6 GCP, GDPR, and HIPAA and is ISO 270001 and ISO 9001 certified. The participants will be identified by a coded ID number to maintain participant confidentiality. The identification fill will be stored separately in a password-protected folder in the hospital’s network. Only members of the research team can access the Castor EDC and the identification file.

### Plans for collection, laboratory evaluation, and storage of biological specimens for genetic or molecular analysis in this trial/future use {33}

Blood samples will be primarily collected via the intravenous line which is already in place (due to surgery). However, when not possible, the samples will be collected via venipuncture. In every patient, several blood samples will be taken. One part of the samples will be directly analyzed by the lab (e.g., ROTEM, coagulation etc.); the other part of the sample will be processed (by the standard operation procedure of the clinical chemistry lab) and stored in − 80 freezers for a more detailed analysis of the coagulation (e.g., global fibrinolysis) and for future analysis and projects. The samples will be kept for a maximal of 5 years after the completion of the study, after which the samples will be destroyed. The other data will be kept for a total of 15 years. All patients give specific written informed consent for the storage of the biological specimens.

## Statistical methods

### Statistical methods for primary and secondary outcomes {20a}

Blood loss will be tested for normal distribution; if normally distributed, the mean with standard deviation will be reported, and differences between the two groups will be tested using Student’s *T*-test. Otherwise, a median with a 25–75% confidence interval will be reported and differences will be tested using a Mann-Whitney *U* test. For secondary outcome parameters, continuous variables (i.e., volume of blood loss, extent of hyperfibrinolysis) will be tested for normal distribution. If they are normally distributed, the mean with standard deviation will be reported and differences will be tested using Student’s *T*-test. Otherwise, a median with a 25–75% confidence interval will be reported, and differences will be tested using a Mann-Whitney *U* test. Binomial data will be reported using percentages, and differences will be tested using chi-squared tests.

### Interim analyses {21b}

In consultation with the Medical Ethics committee, there is no interim analysis planned for this trial due to a low risk for patients and small sample size with an expected short inclusion time due to the number of expected participants.

### Methods for additional analyses (e.g., subgroup analyses) {20b}

This is not applicable. There is no subgroup analysis planned with regard to primary or secondary outcome.

### Methods in analysis to handle protocol non-adherence and any statistical methods to handle missing data {20c}

The data will be analyzed using an intention-to-treat method without methods to handle missing data.

### Plans to give access to the full protocol, participant-level data, and statistical code {31c}

After completion, analysis, and publication, the anonymized participant-level dataset and statistical code will be available on reasonable request with the principal investigator.

## Oversight and monitoring

### Composition of the coordinating center and trial steering committee {5d}

The Maasstad Hospital is the coordinating center and meets on a weekly basis with informal day-to-day contact. The coordinator center will have structured monthly meetings with the participating centers to monitor progress and inclusion and is available for day-to-day contact for knowledge and organizational support.

### Composition of the data monitoring committee, its role and reporting structure {21a}

This is not applicable. There is no data monitoring committee formed for this study in consultation with the Medical Ethics Committee as this is a trial with limited risk for the participants with a relatively small sample size and a relatively short, expected inclusion period.

### Adverse event reporting and harms {22}

Adverse event (AE), serious adverse events (SAE), and suspected unexpected serious adverse reactions (SUSAR) are monitored and recorded after a patient provides informed consent. Adverse events reported spontaneously by the subject or observed during a routine consultation examination by the investigator, or his staff will be recorded. Adverse events regularly associated with surgery or burn treatment will not be monitored: nausea/vomiting, pain, skin rash of surgery site, diarrhea, edema, hypo-/hypertension, psychosis, delirium. In conformation with the Medical Research Involving Human Subjects ACT, SAE will be reported to the Medical Research Ethics Committee (MREC) within 7 days after first knowledge for SAEs leading to death or that are life threatening and for other SAEs within 15 days of first knowledge. SUSARs will be reported twice yearly as a line list to the MREC. Three months after the intervention, the electronic health record and the participant will be consulted to evaluate possible cardiopulmonary of neurologic complications.

### Frequency and plans for auditing trial conduct {23}

The study and each individual study site will be monitored by the scientific bureau of the primary investigation site according to the requirements of the Medical Research Involving Human Subjects ACT.

### Plans for communicating important protocol amendments to relevant parties (e.g., trial participants, ethical committees) {25}

A “substantial amendment” is defined as an amendment to the terms of the METC application, or to the protocol or any other supporting documentation, that is likely to affect to a significant degree:

- The safety or physical or mental integrity of the subjects of the trial;
- The scientific value of the trial;
- The conduct or management of the trial; or
- The quality or safety of any intervention used in the trial.

All substantial amendments will be notified to the METC and to the competent authority.

Non-substantial amendments will not be notified to the accredited METC and the competent authority but will be recorded and filed by the sponsor.

### Dissemination plans {31a}

The data will be analyzed and reported study wide. The primary outcome parameter, possibly in combination with secondary parameters, will be reported within 1 year after completion of recruitment, in a scientific publication, irrespective of statistical significance.

## Discussion

This protocol of a randomized controlled trial aims to investigate the efficacy of Tranexamic acid in reducing blood loss during burn excisional surgery. Furthermore, the study aims to clarify the coagulation status after burn trauma and during the surgical process. Burn patients, already at risk for anemia, can possibly be treated using tranexamic acid to limit blood loss and prevent or lower the transfusion need during the surgical excision of burn wounds. Also, clarifying the status of the coagulation during excisional surgery can give us more insight in the best window for surgery after burn trauma.

## Trial status

The current protocol version is 2.2.2 September 2023. Inclusion started in December 2021 and is expected to finish in December 2025.

## Data Availability

All member of the study team, including the principal investigator, have access to the full trial dataset. The other investigation sites can access the data by a request to the main study team.

## Abbreviations

AE: Adverse events
BSA: Body surface area
DIS: Diffuse intravascular coagulation
ICU: Intensive care unit
ROTEM: Rotational thromboelastometry
MREC: Medical Research Ethics Committee
MEC-U: Medical Ethics Committees United
SAE: Serious adverse events
SOP: Standard operating procedure
SUSAR: Suspected unexpected serious adverse reactions
TEG: Thromboelastography
